# Prediction of cancer progression in a group of 73 gastric cancer patients by circulating cell-free DNA

**DOI:** 10.1186/s12885-016-2977-7

**Published:** 2016-12-09

**Authors:** Wang-Yang Pu, Rong Zhang, Li Xiao, Yong-You Wu, Wei Gong, Xiao-Dong Lv, Feng-Yun Zhong, Zhi-Xiang Zhuang, Xu-Ming Bai, Kai Li, Chun-Gen Xing

**Affiliations:** 1 Department of Oncology, The Second Affiliated Hospital of Soochow University, Suzhou, 215004 China; 2 Department of Obstetrics and Gynecology, The Second Affiliated Hospital of Soochow University, Suzhou, 215004 China; 3 Molecular Medicine Center, The Second Affiliated Hospital of Soochow University, Suzhou, 215004 China; 4 Department of General Surgery, The Second Affiliated Hospital of Soochow University, Suzhou, 215004 China; 5 Department of Interventional Radiology, The Second Affiliated Hospital of Soochow University, Suzhou, 215004 China; 6 Department of Molecular Diagnostics and Biopharmaceutics, College of Pharmaceutical Science, Soochow University, Suzhou, 215004 China

**Keywords:** Gastric cancer, Circulating cell-free DNA, DNA integrity, Alu-qPCR

## Abstract

**Background:**

Circulating cell-free DNA (ccf-DNA) in plasma may contain both specific and non-specific of tumor markers. The concentration and integrity of ccf-DNA may be clinical useful for detecting and predicting cancer progression.

**Methods:**

Plasma samples from 40 healthy controls and 73 patients with gastric cancers (two stage 0, 17 stage I, 11 stage II, 33 stage III, and 10 stage IV according to American Joint Committee on Cancer stage) were assessed respectively. qPCR targeting the Alu repeats was performed using two different sets of primers amplifying the long and short segments. DNA integrity was calculated as a ratio of the long to the short fragments of Alu repeats.

**Results:**

Plasma DNA concentration was significantly higher in patients with stage III and IV gastric cancers than in healthy controls (*p* = 0.028 and 0.029 respectively). The receiver operating characteristic (ROC) curve for discriminating patients with stage III and IV gastric cancers from healthy controls had an area under the curve (AUC) of 0.744 (95% CI, 0.64 to 0.85). Circulating cell-free DNA concentration increased within 21 days following surgery and dropped by 3 months after surgery.

**Conclusions:**

Concentration of ccf-DNA is a promising molecular marker for assessing gastric cancer progression.

**Trial registration:**

Current Controlled Trials ChiCTR-DDT-12002848, 8 October 2012.

**Electronic supplementary material:**

The online version of this article (doi:10.1186/s12885-016-2977-7) contains supplementary material, which is available to authorized users.

## Background

Gastric cancer is the second most common cancer and the fourth leading cause of cancer death [1]. The most significant prognostic indicator is TNM stage [2]. Diagnostic imaging methods such as endoscopic ultrasound or computed tomography are effective for detection of tumor depth, lymph node (LN) and distant metastasis when they are relatively large. However, there is no clinically established blood test which has the predictive ability to determine gastric cancer for their clinical stages. Therefore, the development of a preoperative blood test for assessment of clinical stage of gastric cancer is desired.

Circulating cell-free DNA in blood is a promising biomarker for detection, monitoring, and prognostic prediction of malignant tumor [3]. Concentration of ccf-DNA is influenced by the presence of cancers [4–6]. Methylated DNA detected in ccf-DNA has demonstrated diagnostic and prognostic potential [7, 8]. Circulating tumor DNA has been suggested correlation with surgical resection and disease progression of gastric cancer [9]. It was showed that integrity of ccf-DNA calculated as a ratio of the long to the short DNA fragments is higher in some types of cancers [10–13]. In healthy individuals, ccf-DNA is mainly originated from apoptotic cells which usually release 185 to 200 base pair length of DNA fragments [14]. In contrast, ccf-DNA released from tumor cells varies in size because of pathologic cell death in malignant tumors [15]. Therefore, ccf-DNA concentration and ccf-DNA integrity may have potential for tumor detection and prognostic prediction.

In this study, we measured the concentration of ccf-DNA by quantitative real-time PCR of Alu repeats on 113 plasma samples from gastric cancer patients and healthy controls and analyze the relationship between the ccf-DNA results and clinicopathological findings to assess diagnostic and prognostic values of these genetic markers of gastric cancer.

## Methods

### Collection of plasma samples and clinical information

Plasma samples from 73 patients suffering from gastric cancers and 40 healthy volunteers were assessed. Blood samples were draw before antitumor therapy, within 1–3 weeks after surgery, and during the follow-up period. Staging was based on postoperative histopathology findings for stage 0 to III, and imaging diagnoses were used for stage IV. Patients included were those who had treated in 2013 and 2014 at the Second Affiliated Hospital of Soochow University. The eligibility criteria included adenocarcinoma of stomach and age 18–78 years. Patients with second malignancies, connective tissue disease, and pregnancy were excluded.

### Plasma preparation for Alu-qPCR

Six ml of blood was collected in an EDTA-containing tube, stored at 4 °C, and processed within 6 h. The blood samples were centrifugation at 3000 rpm for 10 min at room temperature to remove the cellular components. Two ml of plasma were cryopreserved at −80 °C until use. DNA was purified from 2 ml of plasma using QIAamp**®** circulating nucleic acid kit according to manufacturer’s instruction.

### Quantitative PCR of Alu repeats

qPCR was performed using two different primer sets for Alu repeats, amplifying 115 bp short and 219 bp long products. The primer set for the 115 bp amplicon (Alu115) amplified both the short and long DNA fragments, and the primer set for the 219 bp amplicon (Alu219) only amplified long DNA fragments. The sequences of the Alu115 primers were forward: 5’-CCTGAGGTCAGGAGTTCGAG-3’ and reverse: 5’-CCCGAGTAGCTGGGATTACA-3’ as described previously [10], the sequences of the Alu219 primers were forward: 5’- CACGCCTGTAATCCCAGCACTTT-3’ and reverse: 5’-CACGCCTGTAATCCCAGCACTTT-3’. The primers Alu219 are newly designed. Alu115-qPCR values represent the total amount of ccf-DNA. Alu219-qPCR results represent amounts of DNA released from tumor cells. DNA integrity index is calculated as the ratio of qPCR-results (Alu219-qPCR and Alu115-qPCR).

The reaction mixture for each Alu-qPCR consisted of 1 μl DNA template, 0.5 μl of the forward and reverse primers, 12.5 μl Quantifast sybr green PCR mix (Qiagen, Germany) and 10.5 μl RNase-free water in a total 25 μl volume. Real-time PCR amplification was performed with precycling heat activation of DNA polymerase at 95 °C for 5 min, followed 40 cycling of denaturation at 95 °C for 20 s, annealing and extension at 65 °C for 30 s using Bio-Rad CFX96 PCR. The absolute equivalent amount of DNA in each sample was calculated according to a standard curve obtained with serial dilutions (37.5 ng-0.375 pg) of prepared genomic DNA from peripheral blood leukocytes of healthy controls. A negative control (without template) was contained in each plate. All qPCR assays were analyzed without knowledge of specimen identity. Each assay was carried out in duplicates.

### Statistical analysis

The Mann-whiney *U*-test was used to compare Alu115 or Alu219/Alu115 between groups of gastric cancers and healthy controls. Kruskal-wallis H-test was used for multiple comparisons between the groups. Receiver-operating characteristic curve and area under the ROC curve were used to assess the diagnosis value of using Alu115 and Alu219/Alu115 for gastric cancers. Statistical analysis were performed using SAS software and results were considered statistically significant at *p* < 0.05 (two tailed).

## Results

### Evaluation of qPCR for measuring DNA concentration

To evaluate the performance of Alu-qPCR for quantification of ccf-DNA, we tested the specificity of the two primer sets for conventional PCR and then monitored the efficiencies and qualities of amplification by real-time PCR of a 10-fold serial dilution of genomic DNA exact from peripheral blood leukocytes of healthy control. Agarose gel electrophoresis of PCR products obtained with the Alu115 and Alu219 primer sets conformed that the target sequence was specifically amplified without major aberrant bands (Additional file 1: Figure S1).

### Clinical and pathologic characteristics of gastric cancers

The mean age was 49.68 ± 11.74 (standard deviation) years for 40 healthy controls and 61.49 ± 10.18 years for 73 patients with gastric cancers. In 10 patients with stage IV gastric cancer, one had liver and lung metastases, two had lung metastases, two had peritoneal metastases, two had para-aortic lymph node metastases, and three had liver metastases. Table 1 shows AJCC stage and histopathology characteristics of patients whose plasma were sampled preoperatively.

**Table 1:** Alu115 of plasma DNA in subgroups of gastric cancer patients

Variable	patients		Alu115	p
	No.	%		
Sex				
male	46	63.01	783.75 (521.35–1223.63)	0.045
female	27	36.99	1484.50 (414.70–2295.50)	
AJCC primary tumor				
Tis-T1	12	16.44	818.4 (478.45–1732.75)	0.724
T2	12	16.44	966.9 (432–1496.25)	
T3	5	6.85	1153.5 (394.00–1238.75)	
T4	44	60.27	1146.5 (676.7–1878.5)	
AJCC regional lymph nodes				
N0	31	42.47	1079.5 (408.2–1544)	0.362
N1	8	10.96	1281 (1106–4104.25)	
N2	14	19.18	1390.5 (606.65–1949.5)	
N3	20	27.40	925.4 (579.55–2087)	
AJCC distant metastasis				
M0	63	86.30	1079.5 (552.95–1670)	0.275
M1	10	13.70	1666.5 (754.25–2479.5)	
AJCC stage				
0-I	19	26.03	1027.65 (408.2–1935)	0.676
II	11	15.07	1484.5 (400.7–1670)	
III	33	45.20	1065.5 (676.7–1572.5)	
IV	10	13.70	1666.5 (754.25–2479.5)	
Histopathologic grade				
middle -high	19	26.04	906.150 (359.50–1213.00)	0.811
low	41	56.16	786.250 (411.45–1586.25)	
unknow	13	17.81		
Lymphovascular invasion				
positive	18	24.66	1190.00 (551.04–1971.88)	0.653
negative	12	16.44	1146.50 (400.70–1389.50)	
unknow	43	58.90		

### Changes in ccf-DNA in plasma of patients with gastric cancers

Circulating cell-free DNA in patients before antitumor therapy was assessed for their levels and integrity. An Alu115-qPCR value represents the total amount of plasma DNA. The median (IQR 25–75) Alu115-qPCR values in healthy controls and patients with stage 0-I, II, III, and IV gastric cancer were 668.08 (401.18–1212.5), 1027.65 (408.2–1935), 1484.5 (400.7–1670), 1065.5 (676.7–1572.5), and 1666.5 (754.25–2479.5) ng ml^−1^, respectively. The Alu115-qPCR values were significant higher in patients with stage III and IV gastric cancer than in healthy controls (*p* =0.028 and 0.029, respectively). A trend of elevation in stage I and II cancer were observed although it was not significant (*p* = 0.194 and 0.095, respectively) (Additional file 2: Figure S2).

An Alu219-qPCR value represents ccf-DNA concentration in longer sizes of plasma DNA. The median (IQR 25–75) Alu219-qPCR values in the controls and patients with stage 0-I, II, III, and IV gastric cancer were 269.05 (149.85–425.35), 240.45 (184.9–582.1), 384.9 (188.85–714.8), 355.15 (189.4–745.55), and 545.05 (165.4–961.6) ng ml^−1^, respectively. The difference of Alu219-qPCR values between healthy controls and patients with gastric cancer was not significant (*p* =0.119).

The plasma DNA integrity was calculated as the ratio of (Alu219-qPCR value/Alu115-qPCR value) of each sample. The median (IQR 25–75) plasma DNA integrity in healthy controls and patients with stage 0-I, II, III, and IV gastric cancer was 0.43 (0.31–0.61), 0.34 (0.19–0.47), 0.39 (0.22–0.47), 0.39 (0.28–0.53), and 0.38 (0.22–0.52), respectively. There was no difference among these five groups.

The Alu115-qPCR values showed a trend that increased with AJCC stage. The ROC curve of plasma DNA concentration for discriminating patients with stage III and IV gastric cancer from healthy controls had an area under the curve value of 0.744 (95% CI, 0.64 to 0.85; Additional file 3: Figure S3). The ROC curve of plasma DNA concentration for discriminating patients with stage III and IV gastric cancer from patients with stage 0-II gastric cancer had an area under the curve value of 0.565 (95% CI, 0.43 to 0.70).

Plasma DNA concentration was independent of age (*p* =0.3745). In 73 patients with gastric cancer, median plasma DNA concentration was not correlated with depth of tumor invasion (*p* =0.724), lymphovascular invasion (*p* =0.960), and lymph node metastasis (*p* =0.289).

Fifty-eight patients with stage 0-III gastric cancer (*n* = 2, 17, 11, 28, respectively) who had radical surgery were followed up by telephone for 2 years. Tumour metastasis was found in one patient with stage II and 10 patients with stage III. Spearman’s ρ coefficient of DFS with Alu115-qPCR values was 0.007 (*P* = 0.95).

### Circulating cell-free DNA dynamics in patients with gastric cancers undergoing surgery

Postoperative ccf-DNA was assessed in 11 of 73 patients with gastric cancer. The distant metastasis was found during surgery in 2 of 11 patients, and palliative gastrectomy was performed on these two patients. The other nine patients with postoperative AJCC stage I to III (*n* = 3, 1, and 5, respectively) received radical gastrectomy.

Alu115-qPCR values in plasma sampled preoperatively from 11 patients varied from 400.7 to 18890 ng ml^−1^(median = 1544 ng ml^−1^).

Within 3 weeks after surgery, Alu115-qPCR values elevated median 109.9% (IQR 59.89–199.37%) than preoperative in 10 of 11 patients. Only one patient with stage III cancer was found with Alu115-qPCR values dropped.

At 3 to 4 months after surgical intervention, Alu115-qPCR values dropped 19.16% (IQR 16.64–38.47) than preoperative in 10 of the 11 patients (90.91%), and in one patient with stage I cancer, Alu115-qPCR values elevated 127.9% than preoperative. In two patients received palliative gastrectomy, Alu115-qPCR values dropped 1.19 and 24.81%, respectively. One patient who had radical surgery developed peritoneal dissemination in 25 months after operation, and Alu-qPCR values elevated again (Additional file 4: Figure S4).

## Discussion

The present study analyzed ccf-DNA in a group of 73 patients with gastric cancers. Our data documented that ccf-DNA levels were elevated in patients with late stage cancers. Although dynamic changes of ccf-DNA levels and integrities are highly useful in monitoring the effects of therapy and progression of the cancer, limitation in obtaining multiple blood samples and relatively high individual variance shadowed its clinical application presently. Our study identified the ccf-DNA elevated significantly in one patient who had metastasis after operation and adjuvant chemotherapy, suggesting that longitudinal instead of comparing with normal controls may be of more clinical impact. Technically, a new pair of primers of Alu219 was tested with high reproducibility in analysis of ccf-DNA in the present study.

Circulating cell-free DNA is a precious biomarker for cancer not only because it contains the DNA released from tumor cell but also pretty reliable and easy to analyze [16]. Studies have demonstrated that concentration of ccf-DNA elevates in patients with lung cancer, breast cancer, gastric cancer, colorectal cancer, urologic tumor, head and neck tumor, etc. [17]. Comparing to concentration of ccf-DNA, concentration of circulating tumor DNA (ctDNA) can reflect tumor burden more accurately [18]. However, detection of tumor-specific gene mutation is limited by several factors. First, there must be tumor-specific gene mutation genetically identified in primary malignant tumor. Second, the low sensitivity and complexity of assays make them less attractive for clinical use, particularly for patients received surgical removal.

Alu repeats are short interspersed elements, typically 300 nucleotides [19]. The Alu sequence is the most abundant sequence in human genome with about one million copies per genome. It is thus more sensitive for Alu-qPCR method in assess the concentration of ccf-DNA [11].

In this study, we assessed the concentration of ccf-DNA by Alu-qPCR methods. We found that the level of ccf-DNA of gastric cancer is higher than healthy controls, especially in stage III and IV. This suggests the potential application in preoperative stage and a representative for cancer progression.

Some recent studies demonstrated that DNA integrity elevated in many types of cancer [10–13]. However, the study about DNA integrity didn’t show difference between patients with gastric cancer and healthy controls [5]. Our result is identified with previously studies. DNA integrity had no significant difference between patients with gastric cancer and healthy controls. This may be partially related to less ctDNA released to blood stream rather than excreted through digestive track for gastric cancers. Thus, DNA from dead tumor cell into blood dropped. Another possibility is that the death pattern of gastric cancers might be different from some other types of cancers due to their environment of very low pH and high level of proteinase, although this speculation needs to be elucidated by experiments in animal models.

We assessed ccf-DNA dynamics in surgical gastric cancer patients. In 21 days after surgery, in most of the patients, ccf-DNA rised, until 3 months after surgery, ccf-DNA was under the preoperative level, no matter undergoing radical or palliative surgery. In one patient developed distant metastasis after radical surgery, the concentration of ccf-DNA was found to be elevated again demonstrating that detection of change in ccf-DNA may be useful for monitoring of the burden of gastric cancer in patients received surgery.

Technically, the present study tested a new pair of primers to amplify the long fragment in Alu assay. This pair of primers were screening from 6 primers. It amplified 219 bp Alu fragments. This primers had higher efficiency than the previous Alu247 primers [11]. The new high efficient pair of Alu219 primers may be applicable for measurement of DNA integrity.

## Conclusions

In conclusion, the present study observed that ccf-DNA was higher in plasma from patients with advanced gastric cancer than those from healthy controls. Circulating cell free-DNA levels dropped after operation and elevated again at tumor progression. Circulating cell-free DNA may be valuable in monitoring progression and prognosis of gastric cancer. In addition, We also tested a new pair of Alu219 primers that have high efficiency and may be applicable for measurement of DNA integrity.

## Additional files

Additional file 1: Figure S1. Results of agarose gel electrophoresis of PCR products obtained with Alu115(0–4) and Alu219(5–9) primer sets. Concentration of genomic DNA template from (0–4) and (5–9) is 0.00375, 0.0375, 0.375, 3.75 and 37.5 ng ml^−1^. (DOC 115 kb)
Additional file 2: Figure S2. Alu115-qPCR values in plasma from healthy controls and patients with gastric cancer. Horizontal lines indicate the median for each groups. The Alu115-qPCR values were significant higher in patients with stage III and IV cancer than in healthy controls. (DOC 210 kb)
Additional file 3: Figure S3. ROC curve for discriminating gastric cancer with stage III and IV from heathy controls. Area under the curve of Alu115-qPCR values was 0.744 (95% CI, 0.64 to 0.85). (DOC 180 kb)
Additional file 4: Figure S4. Circulating cell-free DNA dynamics in patients with gastric cancers undergoing surgery. (A) Pre- and postoperative plasma Alu115-qPCR values in patients with gastric cancer who underwent radical surgery (*n* = 9) and palliative surgery (*n* = 2). (B) Alu115-qPCR values in one patient who had radical surgery and developed peritoneal dissemination at postoperative 25 months. (DOC 234 kb)

## Abbreviations

AJCC: American Joint Committee on Cancer
AUC: Area under ROC curve
ccf-DNA: Circulating cell-free DNA
CI: Confidence interval
ct-DNA: Circulating tumor DNA
IQR: Interquartile range
LN: Lymph node
ROC: Receiver operating characteristic

## References

[1] Ferlay J, Shin HR, Bray F, Forman D, Mathers C, Parkin DM (2010). Estimates of worldwide burden of cancer in 2008: GLOBOCAN 2008. Int J Cancer.

[2] Marrelli D, Morgagni P, de Manzoni G, Coniglio A, Marchet A, Saragoni L (2012). Prognostic value of the 7th AJCC/UICC TNM classification of noncardia gastric cancer: analysis of a large series from specialized Western centers. Ann Surg.

[3] Schwarzenbach H, Hoon DS, Pantel K (2011). Cell-free nucleic acids as biomarkers in cancer patients. Nat Rev Cancer.

[4] Shapiro B, Chakrabarty M, Cohn EM, Leon SA (1983). Determination of circulating DNA levels in patients with benign or malignant gastrointestinal disease. Cancer.

[5] Sai S, Ichikawa D, Tomita H, Ikoma D, Tani N, Ikoma H (2007). Quantification of plasma cell-free DNA in patients with gastric cancer. Anticancer Res.

[6] Park JL, Kim HJ, Choi BY, Lee HC, Jang HR, Song KS (2012). Quantitative analysis of cell-free DNA in the plasma of gastric cancer patients. Oncol Lett.

[7] Kolesnikova EV, Tamkovich SN, Bryzgunova OE, Shelestyuk PI, Permyakova VI, Vlassov VV (2008). Circulating DNA in the blood of gastric cancer patients. Ann N Y Acad Sci.

[8] Balgkouranidou I, Karayiannakis A, Matthaios D, Bolanaki H, Tripsianis G, Tentes AA (2013). Assessment of SOX17 DNA methylation in cell free DNA from patients with operable gastric cancer. Association with prognostic variables and survival. Clin Chem Lab Med.

[9] Hamakawa T, Kukita Y, Kurokawa Y, Miyazaki Y, Takahashi T, Yamasaki M (2015). Monitoring gastric cancer progression with circulating tumour DNA. Br J Cancer.

[10] Umetani N, Giuliano AE, Hiramatsu SH, Amersi F, Nakagawa T, Martino S (2006). Prediction of breast tumor progression by integrity of free circulating DNA in serum. J Clin Oncol.

[11] Umetani N, Kim J, Hiramatsu S, Reber HA, Hines OJ, Bilchik AJ (2006). Increased integrity of free circulating DNA in sera of patients with colorectal or periampullary cancer: direct quantitative PCR for ALU repeats. Clin Chem.

[12] Hao T, Shi W, Shen X, Qi J, Wu X, Wu Y (2014). Circulating cell-free DNA in serum as a biomarker for diagnosis and prognostic prediction of colorectal cancer. Br J Cancer.

[13] Pinzani P, Salvianti F, Zaccara S, Massi D, De Giorgi V, Pazzagli M (2011). Circulating cell-free DNA in plasma of melanoma patients: qualitative and quantitative considerations. Clin Chim Acta.

[14] Giacona MB, Ruben GC, Iczkowski KA, Roos TB, Porter DM, Sorenson GD (1998). Cell-free DNA in human blood plasma: length measurements in patients with pancreatic cancer and healthy controls. Pancreas.

[15] Jahr S, Hentze H, Englisch S, Hardt D, Fackelmayer FO, Hesch RD (2001). DNA fragments in the blood plasma of cancer patients: quantitations and evidence for their origin from apoptotic and necrotic cells. Cancer Res.

[16] Kaiser J (2010). Keeping tabs on tumor DNA. Science.

[17] Jung K, Fleischhacker M, Rabien A (2010). Cell-free DNA in the blood as a solid tumor biomarker—a critical appraisal of the literature. Clin Chim Acta.

[18] Murtaza M, Dawson S-J, Tsui DW, Gale D, Forshew T, Piskorz AM (2013). Non-invasive analysis of acquired resistance to cancer therapy by sequencing of plasma DNA. Nature.

[19] Deininger P (2011). Alu elements: know the SINEs. Genome Biol.

