# Supplementary figures and images for: Prediction of cancer progression in a group of 73 gastric cancer patients by circulating cell-free DNA
Source: BMC Cancer. 2016 Dec 9;16:943. doi: 10.1186/s12885-016-2977-7 (PMC5148873; doi:10.1186/s12885-016-2977-7)

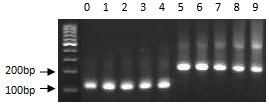

Supplement: Additional file 1: Figure S1. — Results of agarose gel electrophoresis of PCR products obtained with Alu115(0–4) and Alu219(5–9) primer sets. Concentration of genomic DNA template from (0–4) and (5–9) is 0.00375, 0.0375, 0.375, 3.75 and 37.5 ng ml−1. (DOC 115 kb) [file 12885_2016_2977_MOESM1_ESM.doc]

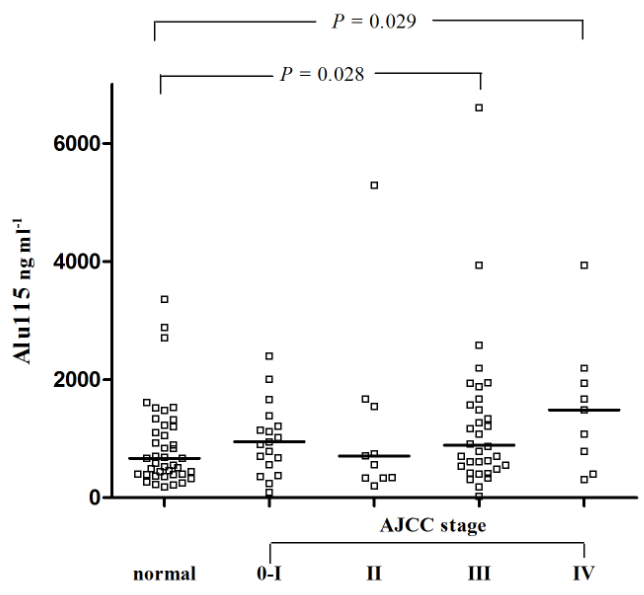

Supplement: Additional file 2: Figure S2. — Alu115-qPCR values in plasma from healthy controls and patients with gastric cancer. Horizontal lines indicate the median for each groups. The Alu115-qPCR values were significant higher in patients with stage III and IV cancer than in healthy controls. (DOC 210 kb) [file 12885_2016_2977_MOESM2_ESM.doc]

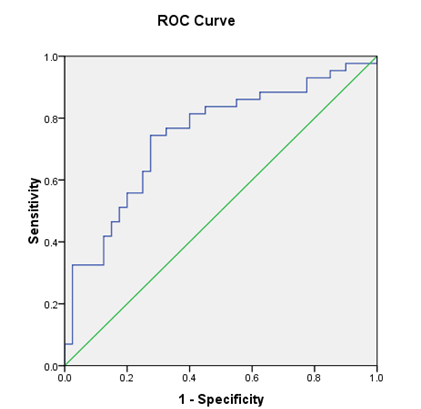

Supplement: Additional file 3: Figure S3. — ROC curve for discriminating gastric cancer with stage III and IV from heathy controls. Area under the curve of Alu115-qPCR values was 0.744 (95% CI, 0.64 to 0.85). (DOC 180 kb) [file 12885_2016_2977_MOESM3_ESM.doc]
