# Supplementary material for: Prediction of cancer progression in a group of 73 gastric cancer patients by circulating cell-free DNA
Source: BMC Cancer. 2016 Dec 9;16:943. doi: 10.1186/s12885-016-2977-7 (PMC5148873; doi:10.1186/s12885-016-2977-7)
Supplement: Supplementary file 2 [file 12885_2016_2977_MOESM4_ESM.doc]

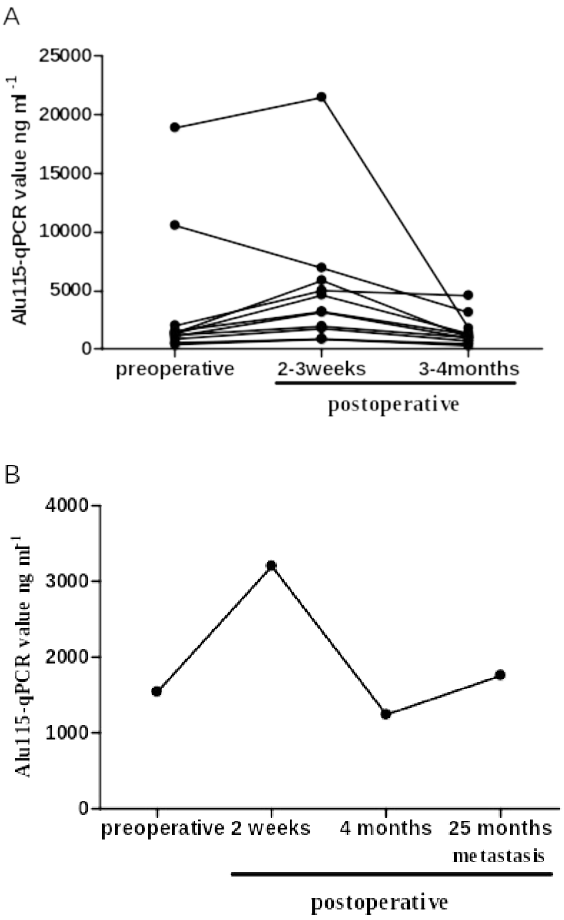
A
Alu115-qPCR value ng ml-1
25000
20000
15000
10000
5000
0
preoperative
2-3weeks
3-4months
postoperative
B
Alu115-qPCR value ng ml-1
4000
3000
2000
1000
0
preoperative
2 weeks
4 months
25 months
metastasis
postoperative
